# Effect of Empty Fruit Brunch reinforcement in PolyButylene-Succinate/Modified Tapioca Starch blend for Agricultural Mulch Films

**DOI:** 10.1038/s41598-020-58278-y

**Published:** 2020-01-24

**Authors:** Rafiqah S. Ayu, Abdan Khalina, Ahmad Saffian Harmaen, Khairul Zaman, N. Mohd Nurrazi, Tawakkal Isma, Ching Hao Lee

**Affiliations:** 1 Laboratory of Biocomposite Technology, INTROP, UPM, Selangor, 43400 Malaysia; 2 Engineering Faculty, UPM, Serdang, 43400 Malaysia; 3 Polycomposite Sdn Bhd, Jalan Maharajalela, Hilir Perak, 36000 Perak Malaysia

**Keywords:** Mechanical engineering, Chemical engineering

## Abstract

In this study, it focused on empty fruit brunch (EFB) fibres reinforcement in polybutylene succinate (PBS) with modified tapioca starch by using hot press technique for the use of agricultural mulch film. Mechanical, morphological and thermal properties were studied. Mechanical analysis showed decreased in values of modulus strength for both tensile and flexural testing for fibres insertion. Higher EFB fibre contents in films resulted lower mechanical properties due to poor fibre wetting from insufficient matrix. This has also found evident in SEM micrograph, showing poor interfacial bonding. Water vapour permeability (WVP) shows as higher hydrophilic EFB fibre reinforcement contents, the rate of WVP also increase. Besides this, little or no significant changes on thermal properties for composite films. This is because high thermal stability PBS polymer show its superior thermal properties dominantly. Even though EFB fibres insertion into PBS/tapioca starch biocomposite films have found lower mechanical properties. It successfully reduced the cost of mulch film production without significant changes of thermal performances.

## Introduction

In South East Asia, the issue of non-biodegradable plastic usage have caused severe environmental pollutions (landfills, carbon footprint)^1^. Increased demand in plastic mulch film has been observed from year 2012–2019 (from 4.4 to 7.4 million tonnes) globally^2^. In agricultural land, mulching films covered more than 8000 km^2^, this is a negative consequences on the excessive used of plastic in order to protect horticultures^3^. In addition, disposal of non-biodegradable films from the fields is costly and time consuming. Hence, farmers usually burned the used plastic films and causing severe air pollution^4^. Awareness on environmental pollution was triggered researchers to locate suitable biopolymers to substitute petroleum derived plastics which are non-degradable in nature. Hence, biodegradable plastics films development for mulching films is highly desirable.

In recent years, Poly-butylene-succinate (PBS) has attracted much attention as one of the common biodegradable polymers used on biodegradable plastic mulches^5^. This is due to its good characteristics including low cost, well processability, good thermal stability and chemical resistance. It also has virtuous physical, mechanical properties and biodegradability for wide applications including mulching films^6,7^. Besides, PBS also gain a lot of attentions due to its high molecular weights which can produces various of products. It is targeted to contributes in large market due to its promising properties^8^. PBS can be produced by a two stage polycondensation starting from 1,4-butanediol and succinic acid^9^. Koitabashi (2012) has fabricated mulch films from PBS polymer and observed a good degradation rate by phylloplane fungi on the field^10,11^. This has reduced the disposal works and cleaning cost significantly to the farmers.

On the other hand, biodegradable films based on hydrocolloids such as starch can act as barriers to control the transfer of moisture, oxygen, carbon dioxide and lipids components, thus preventing quality deterioration and it is one of the important requirement for agricultural mulching film^12^. Tapioca starch has categorized in C-type starch, which consists of 18% amylose. It is widely used for film-based products due to low cost and high availability with a wide potential application in packaging, food industry, agricultural^13,14^. Besides, Tapioca starch derived from cassava roots can growth easily^15^. However, native starch has few limitations and needs gelatinization process to prevent it become granule.

In this research, the author uses modified tapioca starch which has good processability compared with native starch. The used of modified tapioca starch also can reduce processing time for compound formation^16^. One study has investigated the processing properties and strength of composite from the blending of polyester with starch^17^. Precise composition of mixing is needed to achieve good properties of composite. Scopel (2016) has produces agricultural mulch film by using starch and degradation releases nitrogen and carbon, which are nutrients for the soil^18^. Decomposition of biobased mulch films increase microbial activity in the soil, thereby enrich the soil nutrient.

Besides, natural fibres from renewable crops or a by-product of agricultural poses a good potential candidates for use in blends and composites^19^. Natural fibres became a trending in reinforcement materials due to its low in cost, density, high availability, eco-friendly, non-toxic, flexible, renewable and easy to process^20^. These have made it highly compatible in film applications with biopolymers. Palm oil is the most widely traded vegetable oil in the world and the demands is continues breaking the records every year^21^. Malaysia is one of the major quality palm oil exporters and at the same time it is one of the important agriculture and economy income in Malaysia^22^. However, oil palm is producing about 1.1 tonnes of by-product for each tonne of oil produced, 23% of waste are empty fruit brunch (EFB)^23,24^. The use of EFB reinforcement has reported a cheaper in cost with better thermal properties of composite^25^. The kinematic energy consumption also found lesser than without EFB reinforcement polymer composites. Besides, natural fibre reinforced in starch and polymer films have been studied in previous work and the results demonstrated a high potential to substitute non-biodegradable mulch films^26^. On the other hand, the use of fibre reinforced polymer organic mulch films in agricultural helps to endure and strengthen the mulch film in crop cultivation, as well as help plants to adapt climate changes^27^.

In this research PBS, modified tapioca starch and EFB fibres were used as main materials to form composite for mulches film purpose. There are a lot of researches have been done or conducting on bio-based mulch films by using PBS polymer, starch polymer and EFB fibres. However, there is no previous research conducts on the EFB reinforced in PBS/starch polymer blend to provide fully biodegradable mulch films in agricultural. Apart of this, the EFB by-product and tapioca starch which are extremely low in cost when compared to PBS polymer, helps to reduce the production cost significantly, thereby affordable by low-income agricultural farmers. The effects of EFB fibre reinforcements in PBS/starch blends was studied in this research. Physical, mechanical and thermal characterizations have been done to analysis the suitability as a substitute material for agricultural mulches films.

## Materials and Method

### Materials

The PBS were supplied by PTT Co. Ltd, Thailand. The density of PBS is 1.26 g/cm^3^. Modified tapioca starch in form of powder was obtained from PT Starch solution in Indonesia. The viscosities of the starch are 5.5Cp. Meanwhile, oil palm fibres were collected from Polycomposite Sdn Bhd at Labu Negeri Sembilan. The scientific name of oil palm biomass is Elaeis guineensis.

### Sample preparation

To avoid excessive hydrolysis from PBS, the pallets are first dried in an oven at 80 °C. After that it was melted with modified tapioca starch and EFB fibre in a counter rotating internal mixer (Brabender) for 10 minutes at 115 °C with rotation of 60 rpm until the compound turns homogenous. The film formulations have been listed in Table 1. Then, the compound will be transformed into small pieces by using crusher machine. Lastly, the granule mixture will be place and molded under hot press for 4 minutes at 115 °C, followed by 3 minutes of cooling period.

**Table 1:** Composition of PBS, Modified Tapioca starch and EFB Fibres.

Name	PBS + Modified Tapioca Starch Matrix (%)^a^	EFB Fibre (%)
P/EFB (70:30)	70	30
P/EFB (60:40)	60	40
P/EFB (50:50)	50	50

^a^50:50 ratio of PBS and modified tapioca starch in each matrix combinations.

### Characterization

#### Mechanical testing

The tensile test was carried out by using a 10kN Instron universal tester follows the standard ASTM D638 with 50 mm/min strain rate. On the other hand, the flexural testing was conducted according to standard ASTM 790 with 40 mm support span. For both tests, five samples were replicated.

#### Scanning electron microscopy (SEM)

An accelerating voltage of 15 kV in field emission scanning electron microscopy (FESEM) micrograph were taken using Hitachi S-3400N with energy dispersive X-ray (EDX) was applied to investigates surface condition of EFB reinforced PBS/starch films.

#### Thermal analysis

Thermogravimetric analysis was done by TA Instruments Q500 thermogravimetric analyzer, TGA. 20 mL/min of nitrogen flow was set throughout the characterization period. 5–10 mg of specimen was heated in the range of 25–580 °C with 10 °C/min heating rate, to study the mass loss profile.

#### Water vapour permeability (WVP)

The films water vapour permeability (WVP) was measured using a modified ASTM E-96 standard method (ASTM, 1990). The test cup was filled with 6 ml of distilled water and the film sample was tightly fixed over the test cup opening with a rubber gasket. The relative humidity (RH) and temperature conditions of the cups were controlled at 50 ± 5% and 23 ± 2 °C, respectively. The weight of test cups was recorded at a time interval of 1 h for 9 h. WVP was calculated using equation below:

$${\rm{WVP}}=\frac{({\rm{amount}}\,{\rm{of}}\,{\rm{permeant}}\,({\rm{g}})/{\rm{time}}\,({\rm{s}}))\times {\rm{film}}\,{\rm{thickness}}\,({\rm{mm}})}{{\rm{film}}\,{\rm{area}}\,{({\rm{m}}}^{2})\,{\rm{pressure}}\,{\rm{difference}}\,({\rm{kPa}})}$$

## Result and Discussion

### Mechanical analysis

**Figure 1:**
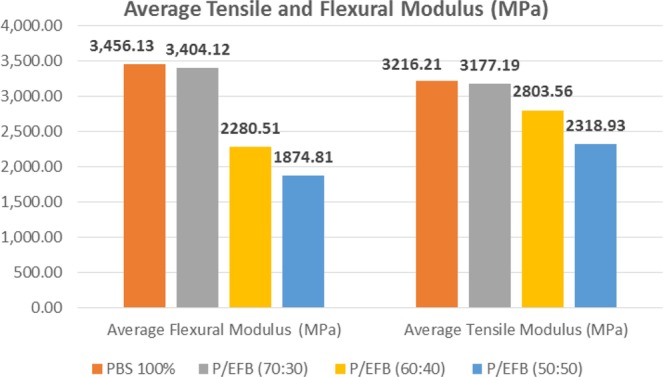
Average Flexural and Tensile Modulus of composite.

**Figure 2:**
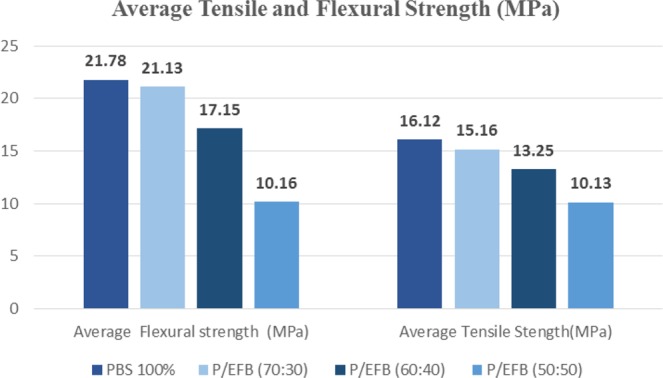
Average Flexural and Tensile Strength of composite.

The composite was prepared with fixed composition of starch and PBS with different volume content of EFB fibres. Mechanical properties of composites are presented in Figs. 1 and 2. It was shown that tensile modulus was reduced as more fibres in composites. All readings (tensile modulus, tensile strength, flexural modulus and flexural strength) have found significantly reduced at 40 wt% of fibre reinforcements. This could be explained by the additional of fibres resulted in insufficient fibre wetting, consequently lowered down load transfer mechanism^28^. On the other hand, poor interfacial adhesion between fibre and matrix creating voids in specimen. This have created stress concentration spots causing the specimen to fail before its maximum load capacity^29,30^. Hence, the composite strength were decrease as increase the percentage of EFB fibre fillers^31^. Besides, lignocellulosic materials in EFB fibre have polar hydroxyl groups at its surface have difficulty in forming well-bonded interface with non-polar PBS matrix. Thus, further resulted in low interfacial bonding strength^32^.

On the other hand, biodegradable polymers having reported a low glass transition temperature (Tg) (40–60 °C) in previous research which causing low strength performances and narrow service temperature^33^. Besides, absent of cross-linked agent or plasticizer is one of the important key factors that causing low in properties. The application of plasticizer helps to reduce the brittleness of starch, consequently having higher flexibility of specimen^34^. In conclusion, insertion fibres with polymer have reduced film mechanical performances.

### Thermal analysis

**Figure 3:**
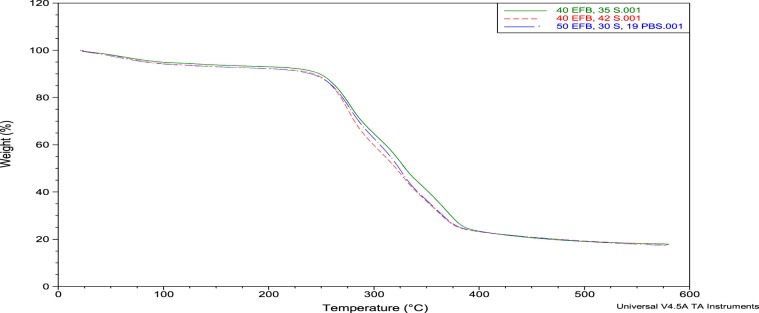
TGA curves of P/EFB composite.

Thermal characterization is one of the methods to understand how the product work under specific working temperature. Table 2 listed the mass lost in every stage with peak temperature and it has plotted in Fig. 3. Thermal decomposition of the composite takes place in the temperature range of 25 to 580 °C and observed in three stages due to the insertion of natural fibres. The initial thermal degradation peak recorded at around 100 °C and it corresponds to moisture removal^35^. Besides, second stage at 250 to 350 °C is the degradation of cellulosic and non-cellulosic materials. The third stage (450 to 550 °C) is due to the final decomposition of polymer material and lignin components^36^.

**Table 2:** TGA analysis for polymer and EFB fibres composite.

Sample	First Mass Loss, %	Temperature, °C	Second Mass Loss, %	Temperature, °C	Mass Residual at 580 °C, %
P/EFB (70:30)	89.63	250.12	44.91	340.16	17.94
P/EFB (60:40)	89.50	245.85	47.08	326.31	17.74
P/EFB (50:50)	88.26	250.12	51.14	322.04	17.32

A slight weight loss is recorded between 80 °C to 100 °C, reported to water removal as starches has the tendency to absorb moisture. The mass loss in range of 150 °C to 380 °C is due to decomposition of three major constituents of EFB fibres and starches polymer which are hemicellulose, cellulose and lignin in sequences^37^. Composition of hemicellulose at 220 °C and their decomposition is substantially completed at 315 °C. Cellulose is thermally stable compared with hemicellulose. At third stage, lignin will decomposed^38,39^.

Second degradation peak was responsible to thermal degradation of PBS polymer, which recorded onset temperature at 322.04–340.16 °C. PBS polymer is one of the polyester family members, which having very high thermal stability. At the end of the test, the film maintains a linear mass loss up to 580 °C, where the final residue is only 17.32–17.94% of the original mass. As conclusion, the insertion of EFB fibres recorded a little or no significant changes in thermal properties of the films. This is because high thermal stability PBS polymer has shown its superior thermal properties dominantly.

### Scanning electron microscopy (SEM)

**Figure 4:**
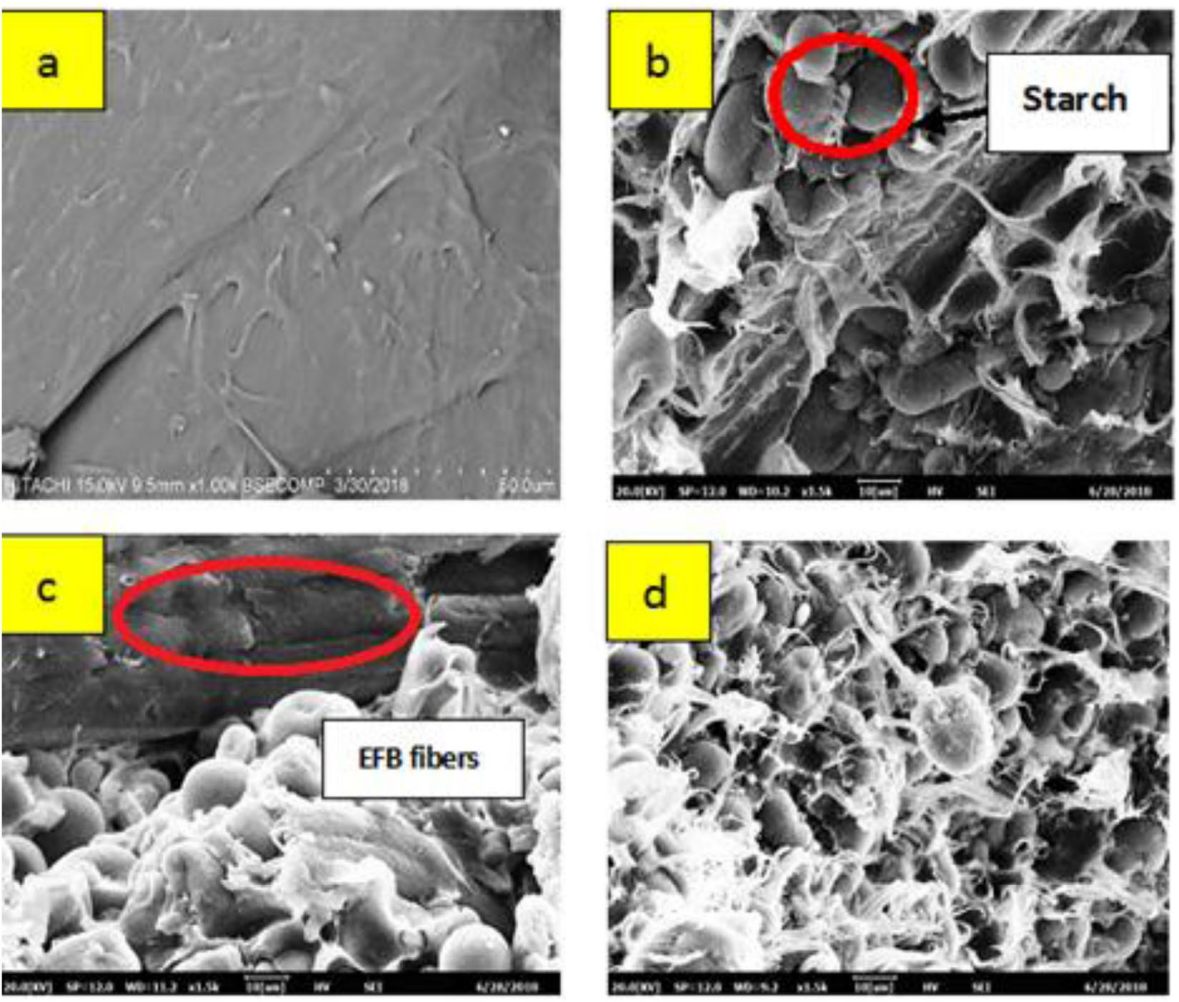
SEM analysis (**a**) Raw PBS (**b**) P/EFB (70/30) (**c**) P/EFB (60/40) (**d**) P/EFB (50/50).

SEM (Fig. 4) was used to study the surface conditions of EFB reinforced PBS/starch biocomposite films. Strength performance of films are directly affected by morphology of composite. Figure 4a shows smooth and regular image of raw PBS. Meanwhile, Fig. 4b–d presents cross section cut of the composite specimen variations (P/EFB(70:30), P/EFB (60:40) and P/EFB (50:50)). Presences of modified tapioca starch shows spherical shape embedded on the PBS surface. However, blending with tapioca starch show weak bonding as stated in previous work^40^.

Figure 4c,d show starch particles and fibres are not well dispersed on the PBS matrix causing weak bonding, indicating fracture happens easily. This have been synchronised with mechanical characterization, which show lower strength performance. One study was reported that fibres reinforcement in polymer cause loose network^41^. Although natural fibres provide strength enhancement to biocomposites, but it may go another way due to incompatibly with hydrophobic polymer^28^. Besides, fibres have high tendency to become more hydrophilic due to presence of polar hydroxyl or other polar groups. Thus, this would resulted in poorer surface adhesion^42^.

### Water vapour permeability (WVP)

WVP is one of the crucial parameters in film production that greatly affects the shelf life of a product. It measures the movement of water vapour molecules passes through the film. Permeates diffusion across a film is influenced by the film structure, film permeability to specific gases or vapour thickness, area, temperature, difference in pressure, or concentration gradient across the film^43^. From the result obtained in Table 3, the trend observed was increased the water vapour permeability of the films after in-cooperating with EFB fibre. WVP reading was increased for higher EFB fibre reinforcements. Film permeability could limit by dispersion of particles in the polymer matrix thus limiting penetration of water molecules thorough the film. However, the presence of fibres which are hydrophilic in nature promotes water molecule sorption, resulted in higher water vapour migration^44^. On the other hand, it needs further study on gaseous permeability on the mulch films surface in the future works.

**Table 3:** WVP Properties of mulch films.

Films	Thickness (mm)	WVP (10^−8^ g mm kPa^−1^ s ^−1^ m^−2^)
PBS 100% (Control)	0.65 ± 0.01	5.71 ± 1.32
P/EFB (70:30)	0.62 ± 0.01	6.21 ± 1.47
P/EFB (60:40)	0.61 ± 0.01	6.36 ± 0.26
P/EFB (50:50)	0.66 ± 0.01	7.02 ± 1.51

## Conclusion

In this study, EFB fibre reinforced in PBS/tapioca starch blends biocomposite films have fabricated. It shows deteriorated strength values when additional EFB reinforcements in biocomposite films. The reason behind this was due to poor fibre wetting for high fibre volume insertion, making the load transfer mechanism ineffective. At the same time, higher void contents were observed. The voids act as stress concentration spot and begin the crack thereby damage the specimen. Besides that, low interfacial bonding is the main key point of low strength performance, recorded from SEM analysis. Polar based EFB have difficulty in forming well bonds with non-polar PBS polymer. All specimens observed little changes in thermal properties because of high thermal stability PBS polymer control the film thermal behaviour. Lastly, WVP reading was increased as increase in percentage of EFB fibre, provide easier water vapour migration during watering section.

As conclusion, we have successfully reduced the cost of mulch film production without significant changes of thermal performances. To further determine the suitability of EFB reinforced PBS/tapioca starch biocomposite films use as agricultural mulch films. Further studies must take from here to investigate the film’s permeability towards gases.

## Data Availability

The datasets generated during and/or analysed during the current study are available from the corresponding author on reasonable request.
